# Postoperative unexplained sigmoid stenosis in a patient with rectal cancer complicated with connective tissue disease: a case report and literature review

**DOI:** 10.3389/fonc.2024.1406098

**Published:** 2024-12-11

**Authors:** Deming Tong, Jian Li, Guangrong Gao, Cheng Zhang

**Affiliations:** Department of General Surgery, General Hospital of Northern Theater Command, Shenyang, China

**Keywords:** rectal cancer (RC), TaTME, CTD (connective tissue disease), anastomotic stenosis, temporary ileostomy

## Abstract

It is well established that host immunity plays a critical role in defending against colorectal cancer (CRC) progression. Connective tissue disease (CTD) encompasses a group of heterogeneous, immune-mediated disorders that present with diverse and often non-specific initial symptoms. Raynaud’s phenomenon is a common feature, complicating early diagnosis. As CTD progresses, it can damage the skin, muscles, and blood vessels and may extend to the lungs, heart, kidneys, and other abdominal organs. Several studies have reported that CTD can lead to intestinal vascular occlusion and related inflammation, but the occurrence of related complications after intestinal surgery has been reported rarely. In this study, an elderly female patient with rectal cancer complicated with CTD was found to have unexplained proximal anastomotic stenosis during an attempt at fistula restoration 3 months after laparoscopy-assisted transanal total mesorectal excision (TaTME) and preventive terminal ileostomy, resulting in fistula failure. This case study aims to serve as a reference for clinicians in their future practice.

## Introduction

**Figure 1:**
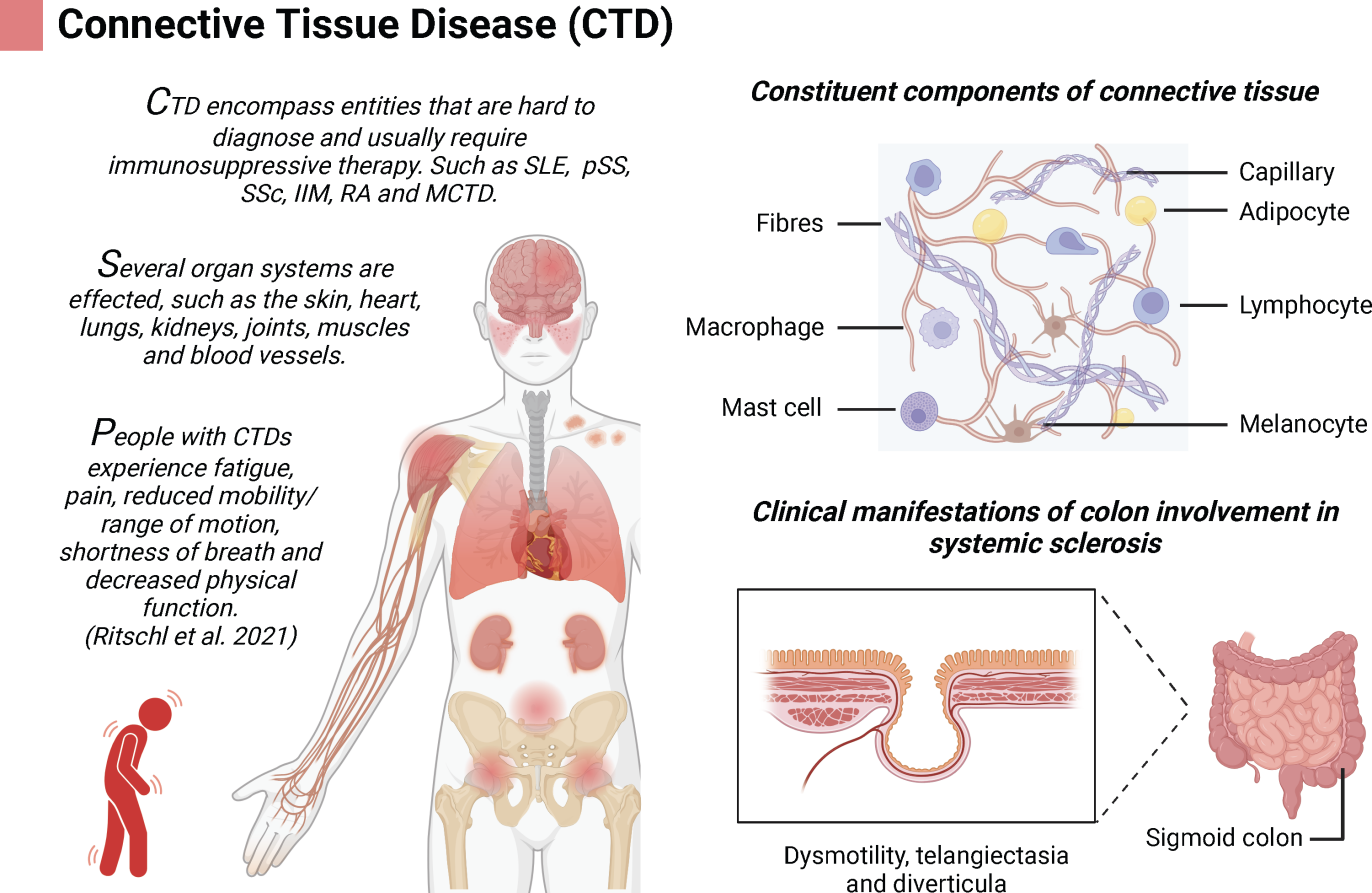
Connective tissue disease (CTD) is complex disease that involves multiple organs; created using BioRender.com.

Host immunity plays a crucial role in preventing the development and progression of cancer. Connective tissue disease (CTD) is a systemic immune disorder that includes manifestations such as rheumatoid arthritis (RA), systemic sclerosis (SSc), systemic lupus erythematosus (SLE), primary Sjogren’s syndrome (pSS), idiopathic inflammatory myopathy (IIM), and mixed connective tissue disease (MCTD) (1, 2). CTD affects multiple organs (skin, heart, lungs, kidneys, joints, muscles, blood vessels, etc.) and leads to various functional impairments (3–5). In addition to CTD itself, secondary vascular inflammatory changes are also a factor of adverse prognosis of the disease, which can occur in different degrees of ischemia, ulceration, and necrosis on the basis of the severity of the involved vessels (6). The intestine is also one of the target organs of CTD patients, especially in patients with SSc. Characteristics of colon involvement in SSc patients include dyskinesia, telangiectasia, and diverticulosis (7). Furthermore, CTD-related vasculitis affects intestinal blood supply, altering intestinal activity, especially postoperatively, potentially exacerbating ischemia (**Figure 1**). Presently, there are few reports on CTD patient recovery and complications after intestinal surgery. This study reported a patient with low rectal cancer with CTD who developed colonic stenosis approximately 2 cm proximal to the anastomosis 3 months after laparoscopy-assisted transanal total mesorectal excision (TaTME) and prophylactic terminal ileostomy. Due to the limited reports on the possible complications of CTD combined with intestinal surgery and the lack of established treatment guidelines, this case report aims to provide valuable insights for future clinical research and practice.

## Case presentation

**Figure 2:**
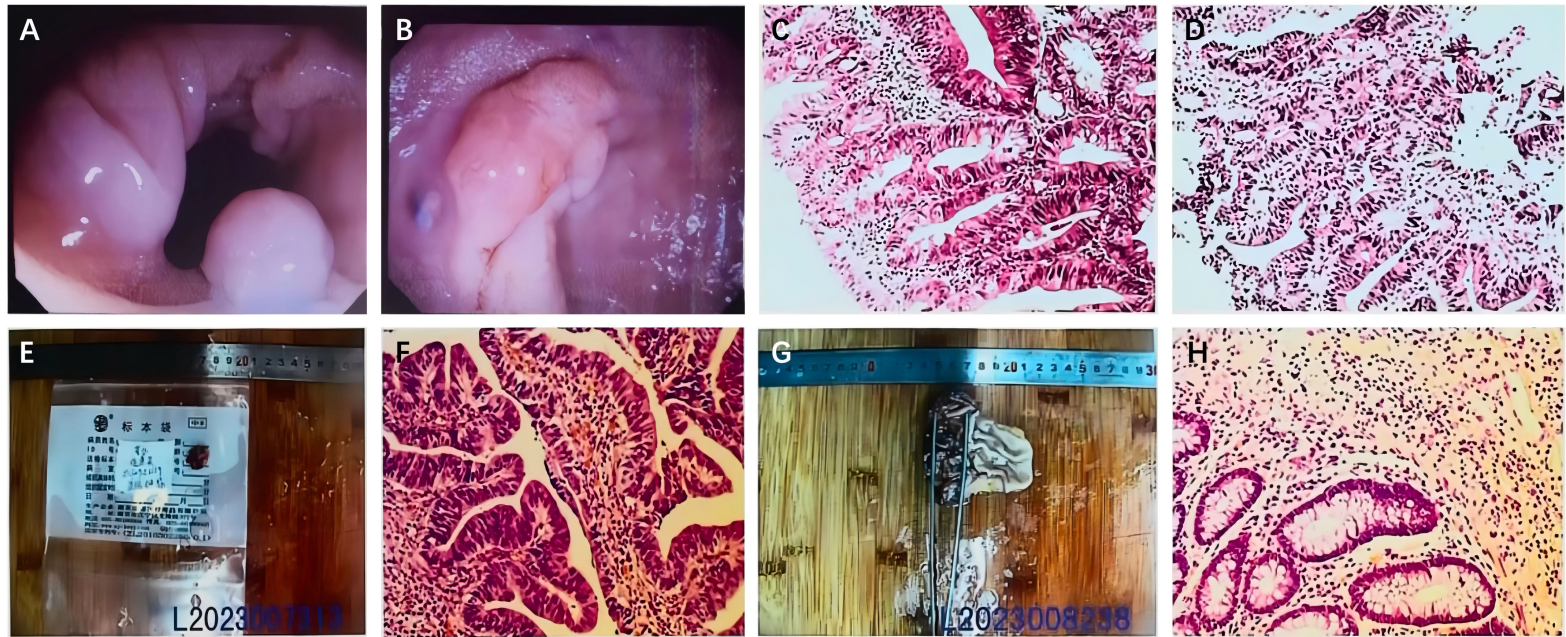
**(A)** A 0.6 * 0.8 cm polyp is seen in the sigmoid colon. **(B)** A 1.5 * 2 cm polyp-like protrusion is seen in the rectum. Pathological findings. **(C)** Tubular adenoma of the sigmoid colon (20 cm). **(D)** Moderately differentiated adenocarcinoma of the rectum (5 cm). **(E, F)** Pathological findings after the initial surgery: moderately differentiated adenocarcinoma of the rectum (5 cm from the anus). **(G, H)** The second operation’s pathological findings: no residual cancer tissue.

A 65-year-old female patient was admitted to our hospital on Mar. 15, 2023, due to rectal cancer discovered during a routine physical examination by fiberoptic colonoscopy with no digestive system symptoms. A polyp approximately 0.6 * 0.8 cm in size was found 20 cm from the anus in the sigmoid colon, which was resected and sent for pathology (**Figure 2A**). Another polypoid lesion, approximately 1.5 * 2 cm in size, was observed 5 cm from the anus. This lesion had a broad base, a sunken surface, a slightly hard texture, and yellow granular changes around it. A specimen was also taken for pathology (**Figure 2B**). The pathological report indicated that the polyp in the sigmoid colon (20 cm) was a colonic tubular adenoma (**Figure 2C**), while the lesion in the rectum (5 cm) was a moderately differentiated adenocarcinoma (**Figure 2D**).

The patient has a medical history of CTD for 2 years. Due to the progressively worsening nature of her condition, she has been treated multiple times in the rheumatology and immunology department of our hospital over the past 2 years. Her long-term oral medications include methylprednisolone 4 mg daily (QD), cyclophosphamide tablets 0.2 mg weekly (QW), and *Tripterygium wilfordii* glycoside tablets 20 mg three times daily (TID). She is currently in a stable condition. Additionally, she has a 3-year history of diabetes mellitus, for which she takes metformin sustained-release tablets orally to manage hypoglycemia, aiming for fasting blood glucose levels of approximately 5.0 mmol/L. The patient also has a 2-year history of hypertension, managed with oral amlodipine besylate tablets to maintain blood pressure within the normal range. The primary diagnoses were as follows: rectal cancer, CTD, Grade II hypertension, and diabetes mellitus. Upon admission, the patient’s physical examination revealed the following findings: body temperature at 36.8°C, pulse rate at 84 bpm, respiratory rate at 18 bpm, and blood pressure at 137/98 mmHg. The physical examination indicated swelling and pain in the knee and finger joints, with restricted movement and morning stiffness lasting over 1 hour. During the digital rectal examination, a mass was palpated on the posterior wall of the rectum, approximately 5 cm from the anal margin. The mass was soft and painless and exhibited good mobility. Laboratory tests showed the following results: red blood cell (RBC) count at 3.51 * 10^12^/L, hemoglobin at 86 g/L, lymphocyte percentage (LY%) at 17.2%, total protein at 61.5 g/L, albumin at 31.1 g/L, C-reactive protein (CRP) at 61.2 mg/L, and glycated hemoglobin (HbA1c) at 7.35%. Antinuclear antibody (ANA) titer was 1:100. Imaging examination included lung CT, revealing interstitial changes in both lungs and mediastinal lymph node enlargement, and abdominal enhanced CT, which showed no thickening of the lower rectal wall or enlargement of lymph nodes in the mesorectum or at the root of mesenteric vessels. Rectoscopy identified a broadly based mass, approximately 1.8 * 1.5 cm in size, with an uplifted surface and good mobility. The mass exhibited no firmness under white light amplification and lacked bleeding points on its surface, and its shape changed after air injection. Endoscopic examination using the NBI International Colorectal Endoscopic (NICE) Classification categorized the mass as Type 2. Considering the patient’s history of connective tissue disease, a rheumatology consultation was obtained for preoperative evaluation. It was recommended to switch the patient’s medication from *T. wilfordii* to hydrocortisone 50 mg Q8h IV during the fasting period.

The preoperative evaluation indicated that the malignant rectal lesions were likely confined to the mucosa or had invaded the superficial submucosa without evidence of distant or regional lymph node metastasis. Preoperative TNM staging was determined to be Tis-1N0M0. On Mar. 23, 2023, diagnostic transanal rectal mass resection was performed under combined spinal-epidural anesthesia (CSEA). The surgery lasted 50 minutes, with an estimated blood loss of approximately 5 mL. The postoperative pathological report revealed moderately differentiated adenocarcinoma of the rectum invading the submucosal layer. No cancer was detected at the base, though the surgical margin could not be evaluated. No clear vascular tumor thrombus or nerve invasion was observed (**Figures 2E, F**). Given the tumor’s submucosal invasion and the indeterminate status of the surgical margin, coupled with the risk of regional lymph node metastasis, further surgical intervention was warranted. To accurately determine the distal resection margin, avoid incomplete distal rectal anastomosis, and enhance surgical safety, laparoscopy-assisted TaTME and a preventive terminal ileostomy were performed under general anesthesia on Mar. 30, 2023. The operation lasted 150 minutes, with a blood loss volume of approximately 50 mL. Oral methylprednisolone at a dose of 4 mg/day was resumed on the second postoperative day. The patient was discharged in a stable condition on Apr. 9, 2023. Postoperative pathology showed no residual cancer tissue in the tumor bed and no lymph node metastasis (0/12) (**Figures 2G, H**).

Under postoperative guidance, the patient consistently performed Kegel exercises to enhance anal sphincter function. At the stoma, defecation was satisfactory, with only a small amount of discharge and gas passing through the anus. Digital rectal examinations conducted at 1 and 2 months post-surgery revealed no signs of anastomotic stenosis.

**Figure 3:**
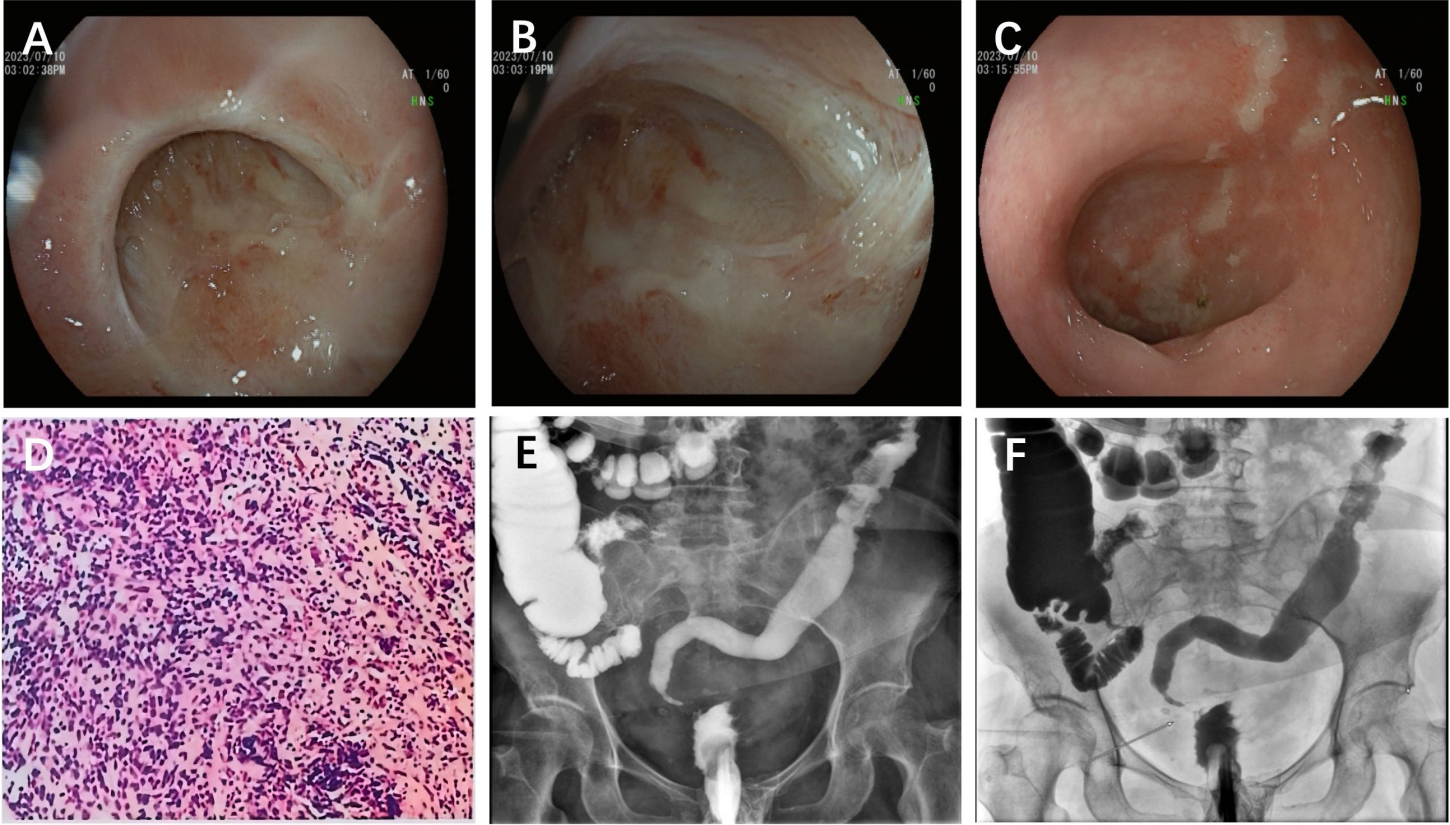
**(A)** Fiberoptic colonoscopy at second admission. The normal anastomosis by fiberoptic colonoscopy through anus. **(B)** A narrow even block intestinal cavity 3 cm from the anus by fiberoptic colonoscopy through anus. **(C)** A narrow intestinal cavity near the sigmoid colon with white moss covered by fiberoptic colonoscopy through abdominal wall. **(D)** Pathological findings of fiberoptic colonoscopy showed inflammatory granulation tissue with a small number of mildly heterotrophic cells. **(E, F)** Iodiography of the lower anal digestive tract showed tubular stenosis of the sigmoid colon.

Three months later, the patient was re-hospitalized for the closure of a temporary ileostomy on Jul. 4, 2023. A digital rectal examination revealed the anastomotic site 3 cm from the anus and a narrowed intestine segment approximately 2 cm above it, indicating potential obstruction. Subsequent colonoscopy showed that the rectal anastomosis was unobstructed, but numerous white, scar-like changes and local annular constrictions with lumen occlusion were observed 2 cm into the lateral cavity. Colonoscopic inspection through the abdominal wall stoma revealed multiple mucosal strips covered with a white plaque near the sigmoid colon, with significant luminal obstruction impeding further endoscopic passage. A biopsy from the ulcer edge (**Figures 3A–C**) revealed necrotic mucosal epithelium and underlying inflammatory granulation tissue with a few mildly atypical cells (**Figure 3D**). Lower gastrointestinal imaging indicated poor elasticity and slightly tubular narrowing of the sigmoid colon, with luminal obliteration. The junction between the sigmoid colon and rectum appeared ring-shaped and narrow, allowing only minimal contrast medium passage (**Figures 3E, F**). The unexplained intestinal stenosis posed significant treatment challenges. While redoing the anastomosis was considered, the patient’s advanced age, existing medical conditions, and nutritional status suggested elevated perioperative risks. Re-anastomosis was complex, raising concerns about whether sufficient colon length remained post-resection. Additionally, the elderly female patient’s compromised anal function and sphincter laxity increased the risk of low anterior resection syndrome (LARS) post-re-anastomosis. After discussion and decision-making, no further stoma reconstruction was performed due to the intestinal stenosis, and the patient was discharged. Relevant laboratory data are presented in **Table 1**.

**Table 1:** The relevant laboratory data of the patient in hospital.

Date (2023)	WBC (*10^9^/L)	NEU (%)	PCT (ng/mL)	CRP (mg/L)	HB (g/L)	P-AlbG (mg/L)	AlbG (g/L)	TP (g/L)	
	3.5–9.5	40.0–75.0	0–0.05	0–3	115–150	180–350	40–55	65–85	Standard range
3.16	6.9	73.7	0.02	24.9	86.0	191.3	33.1	61.5	Be hospitalized
3.24	6.4	74.0	0.02	21.7	81.0	122.4	29.0	57.4	Day 1 after transanal tumor resection
3.26	8.8	80.2	0.02	22.1	87.0	147.6	32.3	63.6	Day 3 after transanal tumor resection
3.31	9.4	81.5	0.02	40.9	87.0	180.4	31.3	62.1	Day 1 after TaTME
4.01	18.7	88.3	0.16	54.9	80.0	110.1	27.7	55.4	Day 2 after TaTME, complicated with intrapulmonary infection
4.02	19.9	92.4	0.42	78.2	76.0	84.5	27.0	53.6	Day 3 after TaTME
4.04	10.4	84.8	0.24	61.7	85.0	109.8	31.3	60.5	Day 5 after TaTME
4.06	9.2	80.7	0.13	32.1	89.0	135.7	29.6	60.7	Day 7 after TaTME
4.09	7.5	75.6	0.05	18.6	88.0	143.1	30.2	61.0	Discharge

WBC, white blood cell; NEU, neutrophils; PCT, procalcitonin; CRP, C-reactive protein; HB, hemoglobin; TP, total protein; TaTME, transanal total mesorectal excision; P-AlbG, prealbumin; AlbG, albumin.

**Figure 4:**
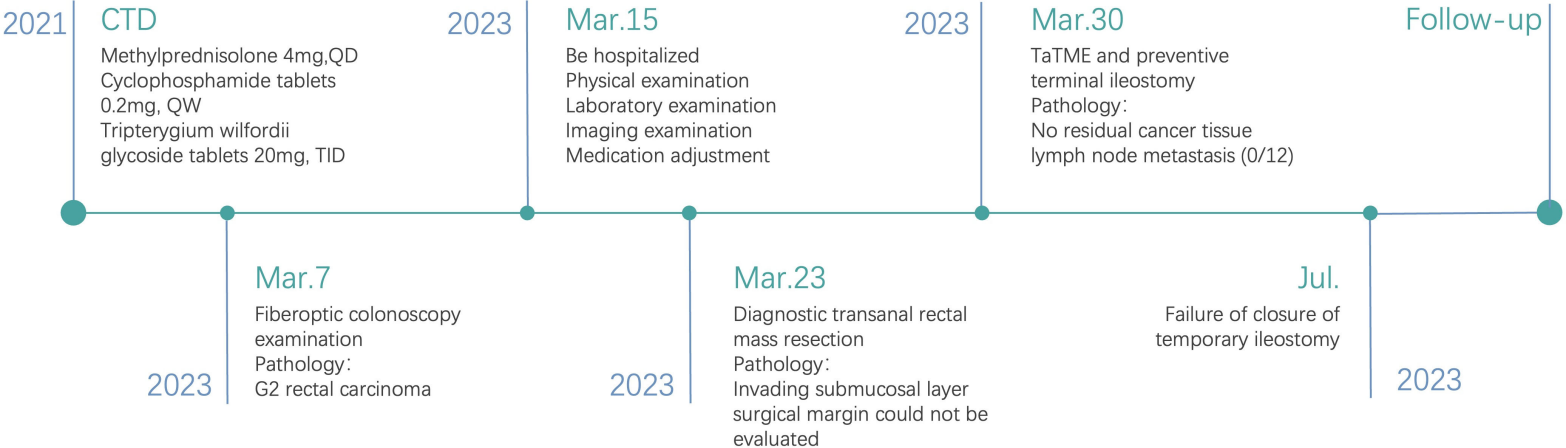
A timeline with relevant data from the episode of care.

Due to unexplained stenosis of the sigmoid colon, the closure of temporary ileostomy was unsuccessful, adversely affecting the patient’s mood. We provided psychological intervention and conducted follow-ups at 6, 9, and 12 months postoperatively. The patient continued medication treatment for connective tissue disease during this period. We observed no signs of tumor recurrence in the postoperative follow-ups, and the stoma was well maintained without needing further reconstruction. The patient’s quality of life remained largely unaffected. No other adverse or unanticipated events occurred. We discussed with the patient the option of additional examinations to assess the alleviation of stenosis for potential ileostomy reversal. However, due to the patient’s age, numerous medical conditions, high risk of complications, and relatively unaffected quality of life, the patient and family members declined further testing. The timeline with relevant data from the episode of care is shown in **Figure 4**.

## Discussion

In this study, we report an elderly female patient with low rectal cancer complicated with CTD who had visited the internal medicine clinic of our hospital for connective tissue disease repeatedly. She was treated with prednisone, cyclophosphamide, and *T. wilfordii* to control the disease as her condition gradually deteriorated. After rectal cancer was found and TaTME was performed, a stricture developed in the colon 2 cm above the anastomosis. To date, no research or case reports have addressed the potential complications of gastrointestinal surgery in patients with CTD, making diagnosis and treatment challenging.

The etiology of intestinal stenosis is frequently linked to ischemic intestinal diseases, often resulting from other medical conditions or medication use, such as ischemic enteritis, diverticulosis, inflammatory bowel disease, intestinal tumors, and radiation enteritis (8). A study utilizing a national registry database in China found that among 1,926 TaTME surgery cases, the overall incidence of complications was 15.4%. Specifically, the incidences of postoperative anastomotic leakage, fever, intestinal obstruction, urinary retention, and incision complications were 5.9%, 4.3%, 2.1%, 1.5%, and 0.8%, respectively. Additionally, 2.8% of the patients experienced pelvic abscess (seven cases), deep vein thrombosis (three cases), cardiopulmonary failure (three cases), and ostomy ischemia or necrosis (three cases). However, proximal anastomotic colonic stenosis was not mentioned (9). The incidence rate of postoperative anastomotic stenosis in TaTME has been reported to be approximately 3.6% (10). Although anastomotic stenosis is often secondary to anastomotic fistula, no such fistula was observed in this patient during the perioperative period, thereby excluding proximal colonic stenosis caused by anastomotic fistula. For this patient, we regularly performed digital rectal examination postoperatively to dilate the anastomosis and prevent stenosis, although proximal stenosis remains rare. A thorough review of the patient’s medical history and perioperative treatment revealed no cause of intestinal stenosis other than CTD. We hypothesize that the surgery disrupted the homeostasis of the local intestinal tissue and exacerbated the inflammatory response associated with CTD, leading to inflammatory narrowing due to altered blood supply to the intestine post-surgery.

CTDs are associated with multiple etiologies, with immune factors being one of the important factors. The presence of autoantibodies in the serum and chronic inflammation in tissues are characteristic manifestations of CTDs, and autoantibodies serve as crucial tools for these conditions (2, 11). The main organ involved in CTDs is the lung (12), but CTDs can also result in gastrointestinal issues such as gastroesophageal reflux, esophageal stenosis, chronic intestinal obstruction, eosinophilic enteritis, and characteristic diverticular changes (7, 13). The occurrence and progression of some CTDs are closely related to intestinal flora. Studies have reported that patients with RA and SLE typically exhibit intestinal flora dysbiosis, characterized by a reduced number of probiotics in the local mucosal flora. The excessive growth of harmful microorganisms and the decreased abundance of beneficial bacteria lead to changes in intestinal mucosal permeability and chronic inflammation (14, 15). This will aggravate the postoperative inflammatory response around the anastomosis, leading to the occurrence of inflammatory stenosis.

In addition to CTD itself, we also discussed the therapeutic drugs associated with CTD. Cyclophosphamide and glucocorticoid are commonly used to treat CTD (16). Cyclophosphamide, a cytotoxic immunosuppressive agent, has no significant toxicity on the gastrointestinal tract. However, Yang et al. reported a case of cyclophosphamide-related severe enteritis involving both the small intestine and the entire colon. The pathology reported severe intestinal mucosal ulcers and granulation tissue formation, which resembled our pathological findings. This case is strongly suspected to be related to cyclophosphamide treatment, but the cause and specific mechanism remain unidentified (17). At the same time, high doses of cyclophosphamide can increase intestinal permeability, alter the balance of intestinal flora, and elevate harmful bacteria counts (18), potentially contributing to inflammation.

Secondary vascular inflammation is commonly observed as a symptom of CTD, with rheumatoid vasculitis (RV) being the most susceptible to secondary vasculitis. Intestinal involvement may present as ischemic colitis (6) and even gangrene. Lupus mesangial vasculitis, one of the most severe gastrointestinal complications secondary to SLE, often leads to gastrointestinal perforation and sepsis. The small intestine (particularly the jejunum and ileum) is most affected (80%–85%), while the rectum is less affected (14%) (6, 19). The patient in this case also presented with Raynaud’s syndrome, which usually affects small arteries in the fingertips and rarely involves the gastrointestinal tract, making the potential inflammatory changes in the intestinal vasculature uncertain. Due to the low location of the tumor, with poor blood supply around the collateral rectal artery, compounded by hypertension, type II diabetes, and other risk factors, the occurrence of ischemic reaction is further aggravated in the presence of secondary vasculitis.

This case report describes the occurrence of sigmoid stenosis above the anastomotic site in a low rectal cancer patient complicated with CTD following surgery. The specific mechanism and risk factors are still unclear. While CTD predominantly targets the lung and is associated with poor prognosis, its impact on the gastrointestinal tract has not been studied widely. Due to the rarity of postoperative complications in CTD patients with concurrent rectal cancer, there is a lack of reliable research conclusions. This report systematically analyzes the potential causes of postoperative proximal anastomotic colonic stenosis in a patient undergoing TaTME surgery complicated with CTD. This case discussion, the first of its kind, expands the scope of CTD-related research in gastrointestinal surgery and aims to guide the clinical management of similar patients.

## Data Availability

The original contributions presented in the study are included in the article/supplementary material. Further inquiries can be directed to the corresponding author.
